# Smoking cessation message testing to inform app-based interventions – an online experiment

**DOI:** 10.21203/rs.3.rs-5707872/v1

**Published:** 2025-03-31

**Authors:** Josef Hamoud, Janardan Devkota, Timothy Regan, Amanda Luken, Joseph Waring, Jasmin (Jiuying) Han, Felix Naughton, Roger Vilardaga, Jonathan Bricker, Carl Latkin, Meghan Moran, Johannes Thrul

## Abstract

**Background:** To improve the efficacy of digital smoking cessation interventions for young adults, intervention messages need to be acceptable and appropriate for this population. The current study compared ratings of smoking cessation and urge reduction messages based on Cognitive Behavioral Therapy (distraction themed) and Acceptance and Commitment Therapy (acceptance themed) in young adults who smoke. **Methods:** A total of 124 intervention messages were rated by an online Qualtrics panel of N=301 diverse young adults who currently smoked tobacco cigarettes (Age M=26.6 years; 54.8% male; 51.5% racial/ethnic minority; 16.9% sexual or gender minority (SGM); 62.5% daily smoking). Each participant rated 10 randomly selected messages (3,010 total message ratings; 24.3 ratings per message) on 5-point scales (higher scores representing more favorable ratings) evaluating quality of content, quality of design, perceived support for coping with smoking urges, and perceived support for quitting smoking. Mixed models examined associations between message category (distraction vs. acceptance), participant level predictors (sociodemographic variables, readiness and motivation to quit, daily smoking, psychological flexibility), and message ratings. **Results:** Overall ratings ranged from M=3.61 (SD=1.25) on support for coping with urges to M=3.90 (SD=1.03) on content, with no differences between distraction and acceptance messages. Male participants gave more favorable ratings on the dimensions of support for coping (p<0.01) and support for quitting (p<0.01). Participants identifying as SGM gave lower ratings for message design (p<0.05). Participants with a graduate degree gave higher ratings on support for coping with urges and support for quitting (both p<0.05). Higher motivation to quit was associated with more favorable scores across all dimensions (all p<0.01). Those smoking daily rated messages as less helpful for coping with urges (p<0.01) and quitting smoking (p<0.05) compared to those smoking non-daily. Few interactions were found between message category distraction vs. acceptance and participant characteristics. **Conclusions:** Distraction and acceptance messages received similar ratings among young adults who smoke cigarettes. Message revisions may be needed to increase appeal to women, SGM, those with lower education, and those less motivated to quit. Messages will be refined and used in an ongoing micro-randomized trial to investigate their real-time impact on smoking urges and behaviors.

## 1. Introduction

Cigarette smoking accounts for close to half a million deaths in the US each year, and is the leading health risk behavior for increased morbidity and mortality.[1,2] While smoking prevalence has generally decreased over recent decades,[3] smoking rates among young adults remain high, and evidence suggests many people who smoke initiate smoking in young adulthood, making this demographic an important group for new smoking cessation interventions.[4–7] Non-daily smoking is common among young adults and ethnic and racial minorities, in particular.[8] Moreover, studies indicate that many non-daily people who smoke are unable to quit on their own and need smoking cessation support.[9] More public health research is needed to understand how to reduce smoking disparities as well as high smoking rates among young adults.[10]

Low socioeconomic status (SES) is an important risk factor for cigarette smoking and reduced success in quitting. Increased smoking rates among members of racial/ethnic minority groups [3,11] and adults of low SES [3,12] are well established. For example, 21.3% of adults with an annual household income <$35k smoke cigarettes compared to 13.7% of adults in the general US population. [3] Similar disparities exist based on education level, such as low education level correlating with increased smoking prevalence among all racial/ethnic groups. [13] . Some of these disparities along lines of race/ethnicity and SES [14,15] can already be observed during the developmental phase of young adulthood, [16] and may endure into later life. Moreover recent evidence suggests disparities among adolescent and young adult members of sexual or gender minority groups (SGM) compared to their heterosexual counterparts, putting them at an increased risk for tobacco use. [17,18] In sum, young adults, who are also often exposed to extensive digital marketing of nicotine products, [19,20] are priority populations for early smoking cessation interventions and sociodemographic risk factors need to be considered in the design of these interventions.

While digital and mobile interventions are in demand, their effective implementation and evaluation in real life settings is lacking.[21] For example, smartphone apps for smoking cessation, a subtype of digital interventions, have become increasingly popular among people who smoke who want to quit. These smartphone apps typically provide tools and resources to help users quit smoking through features like personalized quit plans, progress tracking, urge management, motivational messages, and access to support communities, often enhanced with gamification (elements of game playing, e.g., points, competitions) or reminders that sustain user engagement.[22] As of

2020, English-language smoking cessation apps have been downloaded 33 million times.[23] The low cost and convenience of smartphone-delivered interventions make them an appealing option for many people who smoke, especially those disproportionately affected by the harms of smoking, including those with low income or education, and racial minority subgroups.[24] In addition, the widespread availability of smartphones—96% of US adults 18–29 years old owned one as of 2022—has helped extend the reach of smoking cessation apps to a large number of young adults who may not otherwise have access to cessation services.[25] Thus, smartphone-based interventions hold great promise for reducing the high burden and disparities in smoking-related health risk and mortality. Although many cessation apps do not follow evidence-based guidelines[26–28], some have now been developed using established evidence-based therapies.[22] However, their efficacy is still a major point of investigation in current research.[29–32]

Two key psychotherapy approaches that inform digital smoking cessation interventions and apps are Cognitive Behavioral Therapy (CBT)[33,34] and Acceptance and Commitment Therapy (ACT).[35,36] CBT is focused on changing maladaptive thought and behavior patterns, and has been established as an effective therapy for the treatment of a variety of mental health and substance use disorders, including smoking cessation treatment,[37–40] for in-person as well as digital delivery. One skill taught in CBT is distraction, or shifting attention and awareness to something else when feeling preoccupied with maladaptive or counterproductive thoughts. Thus, distraction may be conceptualized as a coping strategy in CBT. Evidence suggests that distraction strategies can help people who smoke cope with smoking urges;[41] however, more research is needed to investigate perceptions of distraction-themed CBT messages before they can be successfully implemented in digital interventions. In contrast to distraction-themed CBT intervention messages, messages based on a contemporary “third wave” form of CBT called Acceptance and Commitment Therapy (ACT), encourage individuals to pay attention to and build a different relationship with their internal urge-related experiences. For example, one skill emphasized in ACT is acceptance, or being willing to feel and not act on urges to smoke cigarettes, and this concept is called “psychological flexibility”.[42] ACT for smoking cessation has been investigated in previous randomized trials.[43–45] Smoking cessation messages modeled around not only traditional CBT but also ACT theory could provide a wider array of evidence-based and potentially effective digital interventions; however, their perception among diverse groups of young adults needs to be established before efficacy testing.

Important considerations for the implementation of digital smoking cessation interventions concern their design and appropriate content for young adults who smoke. Communication research has demonstrated that tailored messaging is an effective way to convey health information and prompt behavior change, including for smoking cessation.[46–49] There are numerous ways that a message can be tailored. For example, messages can be tailored on content such as message theme; a previous study investigated perceived message effectiveness of e-cigarette warning messages for young adults, showing higher ratings for harm-focused messages compared to social, environmental, or flavor message themes.[50] Messages may also be framed in a way that is tailored to the audience (e.g., gain vs. loss framing).[51,52,53] Additionally, optimizing the color, shapes or the use of signal words may also help increase attention and recall of messages[54] which are important factors contributing to message effects (i.e., as posited in the Message Impact Framework).[55] Previous work on anti-smoking campaigns suggests that perceived effectiveness of message interventions may correlate with their actual effectiveness.[56] However, it is unknown how messages for digital smoking cessation interventions will be rated by young adults from different sociodemographic backgrounds.

In summary, to improve the development and efficacy of novel digital smoking cessation interventions for young adults, including those from diverse backgrounds, intervention messages must meet their needs. While the rating of intervention messages may not align with their actual effectiveness, low rated messages certainly preclude and limit their effectiveness. Therefore, both perceived and actual effectiveness are critical requirements of these digital interventions. The current study used an online panel of young adults who smoke interested in quitting smoking to investigate message ratings of both CBT and ACT-themed intervention messages on different dimensions related to content, design, and perceived message effectiveness to cope with smoking urges and to quit smoking.

### 2. Methods

## 2.1 Procedure

A total of 124 intervention messages were developed by the research team. Intervention messages came from several previous studies,[57–59] were refined internally, and combined with image content from free stock photo websites (Pexels, Unsplash).[60,61] Broadly, distraction messages focused on action-oriented strategies, prompting individuals to redirect their attention away from cravings through specific tasks or behaviors. By promoting active engagement and creative problem-solving, distraction messages aimed at helping individuals break habitual responses to smoking triggers. In contrast, acceptance messages emphasized mindfulness, self-awareness, and reframing cravings as transient and external experiences, encouraging individuals to observe urges without judgment and focus on the present moment. These acceptance messages aimed to empower users by fostering a sense of control through non-resistance and cognitive reframing. See Fig. 1 for intervention message examples. All messages are available on the study OSF page. Study procedures were approved by the Institutional Review Board of the Johns Hopkins Bloomberg School of Public Health.

For the current study, these 124 intervention messages were rated by an online Qualtrics panel of N = 301 diverse young adults (18–30 years) who endorsed current cigarette smoking. Each participant rated 10 randomly selected messages (3,010 total message ratings; 24.3 ratings per message) on dimensions of content, design, perceived support for smoking urge reduction, and perceived support for quitting smoking. The sample size was selected based on recommendations in the existing literature, that suggested 25 evaluations per message to strike a balance between accuracy and efficiency.[62]

### 2.2 Participants

We used Qualtrics panels, an online panel provider for market research, which is increasingly used in health and tobacco research,[63,64] to customize the study sample and facilitate the distribution as well as administration of the survey. The US-based panel included participants who had smoked 100 or more cigarettes in their lifetime, currently smoked cigarettes every day or some days, were between 18 and 30 years old, and were currently trying to quit smoking or intended to quit in the next month. Quotas were set by the research team to ensure diversity of the sample, aiming for approximately 50% male and female sex, 20% Hispanic/Latino ethnicity, and 40% non-White race. These quotas were based on the US Census.[65]

Qualtrics partners with over 20 online sample providers and most samples come from traditional, actively managed, double-opt-in market research panels. Potential participants are randomly selected from sites where users are likely to qualify. Participants' names, addresses and dates of birth are third-party validated (e.g., TrueSample, RelevantID, Verity, etc.). Invitations to participate in the survey are sent through email or on the survey platform with a generic message, a hyperlink to the survey, and the compensation offered. Invitations do not include specific study details to avoid self-selection bias. Since participants are recruited from a variety of sources, incentives vary (e.g., airline points, retail shopping points, cash, or gift cards), but the specific incentive a participant will receive is explicitly stated in the email before participants proceed to the survey link. Digital informed consent was obtained before participants completed the survey.

Qualtrics provides a series of data quality checks, including checking for duplicates, IP addresses, speeding (completion of survey in less than half of median time), bot detection, and straightlining (providing the same answers on 3 or more matrix tables in a survey), among others, before delivering final datasets.

### 2.3 Measures

Participants completed a survey assessing sociodemographic data, including age, sex, SGM status (Response options: Straight; Gay or lesbian; Bisexual; Pansexual; Queer; Transgender, transsexual, or gender non-conforming; Other – specify; recoded to binary variable yes/no), race/ethnicity, education levels, as well as current smoking behavior. For the purposes of reporting message ratings in tables, age was dichotomized into categories 18–25 vs. 26–30, while mixed models included the continuous scale. Smoking behavior measures assessed daily smoking, time to first cigarette (within thirty minutes vs. after 30 minutes),[66] readiness to quit smoking (currently trying to quit; will quit in the next month), and motivation to quit smoking (five-point Likert scale). For the purposes of reporting message ratings in tables, this variable was dichotomized into categories 1–3 (not at all to moderately motivated – low motivation) vs. 4–5 (very or extremely motivated – high motivation), while mixed models included the continuous scale. Psychological flexibility was assessed using the Acceptance and Action Questionnaire – version two (AAQ-II),[67] with higher scores indicating more inflexibility. The AAQ-II measures psychological flexibility, which includes the ability to accept negative experiences, unwanted thoughts, and feelings while remaining engaged in present-moment living and pursuing actions aligned with personal values. Psychological flexibility may be a moderator of message rating of distraction and acceptance messages. Again, for the purposes of reporting message ratings in tables, this variable was dichotomized into categories 7–27 (flexible) vs. 28–49 (inflexible), while mixed models included the continuous scale. A cutoff of 28 or higher has been associated with mental distress in previous work.[67]

Message ratings were assessed across four dimensions: (1) perceived quality of *content* ("How would you rate the content (that is, the words and meaning) of this message?") and (2) perceived quality of *design* ("How would you rate the design (that is, how the message looks) of this message?"), both assessed on a Likert scale from 1 (Very poor) to 5 (Very good). Additional dimensions included: (3) perceived message support for *coping* with smoking urges ("How helpful would this message be to support you in coping with a smoking urge or craving?”), and (4) perceived message support for *quitting* smoking (“How helpful would this message be to support you in quitting or reducing smoking?”), both assessed on a Likert scale from 1 (Not at all helpful) to 5 (Extremely helpful).

### 2.4 Statistical analyses

In descriptive analyses, we examined message ratings by message category (acceptance vs. distraction) and participant characteristics.[62] We used mixed models to examine the relationship between the message category (CBT/distraction vs. ACT/acceptance) and participant level predictors (sociodemographic factors; daily smoking; time to first cigarette; readiness and motivation to quit; psychological flexibility) of message ratings. The model included both fixed and random effects to account for the hierarchical structure of the data, where message ratings were nested within participants. Fixed effects were specified for message category (CBT/distraction vs. ACT/acceptance) and participant characteristics, estimating their average effects across the sample. To account for variability in baseline levels of message ratings across participants, we included a random intercept for participants. The initial mixed models included only main effects. Additional mixed models were estimated to investigate cross-level interaction effects between message category (CBT/distraction vs. ACT/acceptance) and participant level predictors. Separate models were estimated for each interaction effect and controlled for all other predictors.

### 3. Results

#### 3.1 Sample description

Participant (N=301) mean age was 26.57 (SD=2.94) years and 54.8% of participants were male. 16.9% of participants identified as SGM (Table 1). A total of 48.5% identified as Non-Hispanic White, 21.9% as Non-Hispanic Black, and 22.6% as Hispanic. The majority (65.9%) had at least some college education. Regarding smoking behavior, 62.5% of participants reported smoking daily, with 60.8% reporting smoking within thirty minutes of waking up, and 73.7% reported currently trying to quit smoking. The mean rating for motivation to quit smoking was 3.82 (SD=0.93, range 1-5). Psychological flexibility (AAQ-II) mean score was 23.44 (SD=8.22, range 7-39), with 34.6% reporting scores between 28-49, indicating moderate levels of inflexibility.

#### 3.2 Average message ratings

Overall average message ratings were M=3.90 (SD=1.03) on content, M=3.84 (SD=1.06) on design, M=3.61 (SD=1.25) on support for coping, and M=3.62 (SD=1.29) on support for quitting smoking, with little to no differences between distraction and acceptance messages (Figure 2).

Males, participants aged between 26-30 years of age, Hispanic participants and participants with at least a bachelor’s degree gave relatively higher scores on average on most dimensions (Table 2). Participants reporting daily smoking, time to first cigarette at thirty minutes or less, high motivation to quit smoking, or more flexibility gave relatively higher scores across all dimensions compared to their counterparts. Conversely, participants identifying as a SGM or currently trying to quit in the next month averaged lower scores.

#### 3.3 Mixed models

##### 3.3.1 Mixed models predicting message scores based on message and participant characteristics

Mixed models examined main effects of message theme and participant characteristics (Table 3). Distraction messages were rated higher than acceptance messages on the dimension of perceived quality of design ($p<0.05$) and there were no other message category main effects on any of the rating dimensions. Male participants were significantly more likely to report favorable ratings on the dimensions of support for coping ($p<0.01$) and support for quitting ($p<0.01$). Participants identifying as SGM gave significantly lower ratings for message design ($p<0.05$), compared to their non-SGM counterparts. Participants with a graduate degree gave a higher rating on the dimensions of support for coping and support for quitting (both $p<0.05$), compared to those with high school education or less. High motivation to quit was associated with significantly more favorable message scores across all dimensions (all $p<0.01$). Participants who smoked daily rated messages as less helpful to support coping with smoking urges ($p<0.01$) and support for quitting smoking ($p<0.05$), compared to participants who smoked non-daily.

##### 3.3.2 Mixed models predicting message scores with interactions between message and participant characteristics

Few significant interactions were observed between participant characteristics and ratings of distraction- vs. acceptance themed messages.

Participants who identified as SGM gave significantly lower ratings on distraction-themed messages on the dimension of perceived message support for coping with urges compared to their non-SGM counterparts (b=-0.187, SE=0.092, z=-2.02, p=0.043). Regarding racial and ethnic background, compared to Non-Hispanic White participants, Non-Hispanic Black (b=-0.169, SE=0.081, z= 2.09, p=0.037) and Hispanic participants (b=0.198, SE=0.079, z=2.50, p=0.013) gave significantly higher ratings for distraction-themed messages on the dimension of perceived quality of design. Compared to Non-Hispanic White participants, those of other racial or multiracial background gave more favorable ratings for distraction-themed messages on the dimension of perceived message support for coping (b=0.339, SE=0. 136, z=2.48, p=0.013). The only significant interaction between participant education level and message theme was observed among participants with a bachelor's degree, who gave significantly lower ratings for distraction-themed messages on the dimensions of perceived quality of design (b=-0.180, SE=0.084, z=-2.15, p=0.032), perceived message support for coping (b=-0.219, SE=0.09, z=-2.39, p=0.017), and perceived message support for quitting (b=-0.207, SE=0.090, z=-2.28, p=0.022), compared to participants with high school education or less.

### 4. Discussion

The current study recruited an online panel of young adults who smoked and were interested in quitting smoking to evaluate CBT/distraction and ACT/acceptance-themed intervention messages on different dimensions related to content, design, and support for coping with smoking urges, and support for quitting smoking. Our results suggest that CBT/distraction and ACT/acceptance-themed messages were equally well received by our sample of young adults who smoke, reaching similar average ratings across all dimensions. These findings are in line with previous studies comparing the effectiveness of both therapy strategies.[68,69] Overall, our findings are in accordance with previous work that have established the feasibility of both CBT and ACT smartphone-based interventions for smoking cessation,[37–41,43–45] and suggest that both strategies of smoking cessation intervention are similarly well received among young adults who smoke. Although messages of acceptance may be perceived as counterintuitive and different from messaging to avoid urges to smoke based on CBT approaches, our results suggest that the framing and presentation of the acceptance messaging were just as effective as CBT avoidance messaging.

Results of the current study also highlighted sex differences in message ratings and suggest that male participants rated messages more positively than female participants on the dimensions of support for coping with smoking urges and for quitting smoking. These findings align with prior research indicating that biological and psychosocial factors influence smoking behaviors and cessation outcomes differently by sex and gender identity. While women are more likely to adhere to smoking interventions, they experience increased difficulty in cessation.[70–72] [72] Thus, our research provides further evidence for the need of appropriate interventions in the form of tailored message design and content to facilitate coping with smoking urges and support quitting among female young adults who smoke.

Compared to non-SGM participants, those identifying as SGM rated messages lower on the design dimension. Since intervention messages were not tailored to specific subgroups of young adults who smoke cigarettes, including those identifying as SGM, these findings may indicate the need for customized intervention messages to culturally fit this group.[73] There is some evidence that culturally tailored smoking cessation interventions may work better for SGM young adults who smoke compared to non-tailored interventions.[74] However, other research has indicated that while SGM individuals who smoke may prefer smoking cessation interventions that are culturally tailored, untailored interventions show similar effects for SGM people who smoke compared to heterosexual individuals.[75] Future work is needed to determine the efficacy of digital smoking cessation interventions for SGM adults who smoke cigarettes and to investigate the need for cultural tailoring of intervention content.

Regarding effects of education, participants with a graduate degree rated messages significantly more favorable on support for coping with smoking urges and support for quitting smoking compared to participants with a high school degree or less. These findings agree with well-known smoking disparities by education and socioeconomic status.[76,77] Moreover, previous research has demonstrated that among people who smoke, those with less education engage less frequently with traditional smoking cessation aids, despite facing greater exposure to harmful products.[3,11,12] Based on these findings, the development and testing of smoking cessation interventions that are acceptable among people who smoke with low educational attainment and meet their needs should be prioritized. Specifically, future interventions should make sure to engage this population to ensure messages are understandable and appealing, and to make any necessary adaptations.

Our findings showed that participants with a high motivation to quit gave higher message ratings on all dimensions. Conversely, those smoking daily rated messages as less helpful for coping with smoking urges and for quitting. Previous studies have already established the importance of smoker's motivation to quit for the effectiveness of smoking cessation interventions, [78–80] which is also reflected in our results.

Future work may need to improve these smoking intervention messages to not only appeal to those who are already highly motivated to quit, but also to those who report indicators of high nicotine dependence or who may be less motivated to quit. For example, content based on motivational interviewing has been shown to generate good engagement among young adults who smoke and have low motivation to quit in social media delivered interventions.[81,82] Overall, communication research finds that tailored messaging can support smoking cessation and health behavior change more broadly, [47–49,52] and additional tailoring of the messages used in the current study may be needed to improve their efficacy and impact.

#### Limitations

The current study has several limitations. While the online panel we used in the current study allowed us to include various quotas to ensure our participants had diversity regarding sex and race/ethnicity, the study sample may not be representative for all young adults who smoke in the US. For example, our panel did not include a quota for education. Study participants were recruited from different sources with varying incentives, which may have impacted their motivation to diligently complete study procedures. The current study compared different categories of messages (e.g., distraction vs. acceptance) and did not investigate additional intervention message features (e.g., image content) that may have impacted message ratings. Despite evidence from anti-smoking campaigns suggesting that perceived effectiveness correlates with the actual effectiveness of intervention messages to some extent,[56] it is unclear to what degree results of this intervention will be translated into message efficacy to support coping with smoking urges and quitting smoking. In addition, message ratings were aggregated which may have concealed within-individual or within-message variability or consistency. Lastly, messages only addressed cigarette smoking and additional modifications may be needed to address other nicotine and tobacco products popular among young people, which include electronic cigarettes[83] and oral nicotine pouches.[84,85]

### Conclusions

Distraction and acceptance messages to support coping with smoking urges and smoking cessation received similar ratings among young adults who smoked cigarettes. Message revisions may be needed to increase appeal to women, SGM, those with lower education, and those less motivated to quit smoking. As a next step, intervention messages will be tested for efficacy to reduce smoking urges and support smoking cessation in a micro-randomized trial to investigate their real-time impact on behavioral outcomes. This trial is currently in progress and results of the current study helped to select a subset of intervention messages for inclusion. Finally, building on the findings of our current study, future research should also consider collecting and evaluating qualitative feedback and focus-group discussions of intervention messages to further refine and improve digital smoking cessation interventions for priority populations.

### Abbreviations

AAQ-II: Acceptance and Action Questionnaire – version two; ACT: Acceptance and commitment therapy; CBT: Cognitive behavioral therapy; IQR: Interquartile range; M: Mean; NH: Non-Hispanic; OSF: Open Science Framework; SD: Standard Deviation; SES: Socioeconomic status; SGM: Sexual or gender minority; US: United States.

### Declarations

#### Ethics approval and consent to participate

This study adhered to the Declaration of Helsinki and was approved by the Institutional Review Board of the Johns Hopkins Bloomberg School of Public Health. Digital informed consent was obtained before participants completed the survey.

#### Consent for publication

Not Applicable.

#### Data availability

The dataset analyzed for the current study and all intervention messages are available on the project OSF page (https://osf.io/4ux8q/.)

#### Competing interests

Dr. Thrul reports membership on the scientific advisory board of MindCotine, Inc., which offers a smoking cessation program. This arrangement has been reviewed and approved by the Johns Hopkins University in accordance with its conflicts of interest policies. Dr. Moran served as a paid expert witness in litigation sponsored by the Public Health Advocacy Institute against R.J. Reynolds. This arrangement has been reviewed and approved by the Johns Hopkins University in accordance with its conflict of interest policies.

**Funding**

This work was supported by the National Cancer Institute (NCI; R01 CA246590) and the National Institute on Drug Abuse (NIDA; T32 DA007292).

**Contributions**

JH: Writing - Original Draft; Writing - Review & Editing

JD: Data Curation; Formal analysis; Investigation; Writing - Review & Editing

TR: Writing - Review & Editing

AL: Investigation; Writing - Review & Editing

JW: Investigation; Writing - Review & Editing

JH: Writing - Review & Editing

FN: Resources; Writing - Review & Editing

RV: Resources; Writing - Review & Editing

JB: Resources; Writing - Review & Editing

CL: Conceptualization; Methodology; Writing - Review & Editing

MM: Conceptualization; Methodology; Writing - Review & Editing

JT: Conceptualization; Formal analysis; Funding acquisition; Methodology; Supervision; Writing - Original Draft; Writing - Review & Editing

**Acknowledgements**

The opinions and assertions expressed herein are those of the author(s) and do not reflect the official policy or position of the Uniformed Services University of the Health Sciences or the Department of Defense.

## Tables

**Table 1:** Participant characteristics

| | Number (%); M (SD) |
|---|---|
| Age, mean (SD) | 26.57 (2.94) |
| Age categories | |
| 18-25 | 31.89 |
| 26-30 | 68.11 |
| Sex | |
| Female | 45.18 |
| Male | 54.82 |
| Sexual or gender minority | |
| No | 83.06 |
| Yes | 16.94 |
| Race/Ethnicity | |
| Non-Hispanic White | 48.50 |
| Non-Hispanic Black | 21.93 |
| Hispanic | 22.59 |
| Other or multi-racial | 6.98 |
| Education | |
| High school or less | 34.22 |
| Some college | 30.56 |
| Bachelor’s degree | 23.26 |
| Graduate degree | 11.96 |
| Daily smoking | 62.46 |
| Time to first cigarette (30 mins or less) | 60.80 |
| Readiness to quit smoking | |
| Currently trying to quit | 73.75 |
| Will quit in the next month | 26.25 |
| Motivation to quit smoking, mean (SD) | 3.82 (0.93) |
| Motivation to quit smoking categories | |
| Low (1-3) | 32.56 |
| High (4-5) | 67.44 |
| Psychological flexibility (AAQ-II), mean (SD) | 23.44 (8.22) |
| Psychological flexibility (AAQ-II) categories | |

| | |
|---|---|
| Flexible (7-27) | 65.45 |
| Inflexible (28-49) | 34.55 |

**Table 2**: Average ratings for acceptance (N=1,575 ratings) and distraction messages (N=1,434 ratings) on dimensions content, design, coping, and quitting

| | **Content score** (M, SD) | | **Design score** (M, SD) | | **Coping score** (M, SD) | | **Quitting score** (M, SD) | |
|---|---|---|---|---|---|---|---|---|
| Message theme | Acceptance | Distraction | Acceptance | Distraction | Acceptance | Distraction | Acceptance | Distraction |
| **Overall** | 3.90 (1.03) | 3.90 (1.03) | 3.81 (1.07) | 3.88 (1.04) | 3.60 (1.26) | 3.62 (1.25) | 3.60 (1.30) | 3.63 (1.27) |
| **Age** | | | | | | | | |
| 18-25 | 3.84 (1.09) | 3.87 (1.11) | 3.70 (1.12) | 3.81 (1.06) | 3.53 (1.25) | 3.55 (1.26) | 3.50 (1.33) | 3.57 (1.25) |
| 26-30 | 3.93 (1.00) | 3.92 (1.00) | 3.86 (1.04) | 3.91 (1.03) | 3.63 (1.26) | 3.65 (1.24) | 3.65 (1.29) | 3.66 (1.28) |
| **Sex** | | | | | | | | |
| Female | 3.79 (1.07) | 3.74 (1.11) | 3.67 (1.12) | 3.71 (1.13) | 3.35 (1.35) | 3.31 (1.35) | 3.35 (1.39) | 3.30 (1.40) |
| Male | 4.00 (1.00) | 4.04 (0.95) | 3.93 (1.01) | 4.02 (0.94) | 3.81 (1.14) | 3.87 (1.09) | 3.81 (1.19) | 3.90 (1.08) |
| **SGM** | | | | | | | | |
| No | 3.95 (1.02) | 3.98 (1.00) | 3.87 (1.04) | 3.96 (1.00) | 3.66 (1.24) | 3.72 (1.22) | 3.68 (1.29) | 3.74 (1.23) |
| Yes | 3.69 (1.08) | 3.52 (1.14) | 3.51 (1.13) | 3.48 (1.14) | 3.33 (1.29) | 3.10 (1.27) | 3.24 (1.32) | 3.09 (1.32) |
| **Race/Ethnicity** | | | | | | | | |
| Non-Hispanic White | 3.88 (0.99) | 3.89 (1.00) | 3.86 (1.03) | 3.86 (1.03) | 3.63 (1.19) | 3.59 (1.21) | 3.63 (1.23) | 3.63 (1.21) |
| Non-Hispanic Black | 3.82 (1.09) | 3.78 (1.09) | 3.71 (1.10) | 3.78 (1.04) | 3.48 (1.39) | 3.46 (1.36) | 3.45 (1.43) | 3.43 (1.41) |
| Hispanic | 4.08 (1.01) | 4.00 (1.04) | 3.85 (1.07) | 4.00 (1.04) | 3.81 (1.20) | 3.84 (1.15) | 3.83 (1.25) | 3.87 (1.17) |
| Other or multi-racial | 3.78 (1.17) | 4.05 (1.05) | 3.67 (1.14) | 3.95 (1.07) | 3.21 (1.36) | 3.55 (1.37) | 3.20 (1.37) | 3.41 (1.43) |
| **Education** | | | | | | | | |
| High school or less | 3.82 (1.09) | 3.80 (1.05) | 3.69 (1.09) | 3.81 (1.01) | 3.38 (1.34) | 3.44 (1.25) | 3.35 (1.39) | 3.43 (1.29) |
| Some college | 3.76 (1.09) | 3.72 (1.11) | 3.63 (1.12) | 3.69 (1.09) | 3.44 (1.32) | 3.43 (1.32) | 3.42 (1.32) | 3.40 (1.33) |
| Bachelor’s degree | 4.11 (0.88) | 4.17 (0.90) | 4.04 (0.98) | 4.10 (0.97) | 3.87 (1.07) | 3.92 (1.14) | 3.89 (1.16) | 3.97 (1.13) |
| Graduate degree | 4.11 (0.86) | 4.13 (0.91) | 4.17 (0.86) | 4.14 (1.02) | 4.12 (0.90) | 4.03 (1.00) | 4.20 (0.89) | 4.15 (0.99) |
| **Daily smoking** | | | | | | | | |
| Every day | 3.98 (0.99) | 3.93 (1.00) | 3.90 (1.03) | 3.92 (1.01) | 3.75 (1.18) | 3.70 (1.20) | 3.75 (1.23) | 3.72 (1.21) |
| Some days | 3.78 (1,10) | 3.85 (1.09) | 3.66 (1.12) | 3.82 (1.10) | 3.35 (1.34) | 3.48 (1.32) | 3.36 (1.39) | 3.49 (1.35) |
| **Time to first cigarette** | | | | | | | | |
| After 30 minutes | 3.86 (1.05) | 3.86 (1.07) | 3.74 (1.11) | 3.82 (1.09) | 3.46 (1.33) | 3.52 (1.28) | 3.46 (1.37) | 3.49 (1.33) |

| | | | | | | | | |
|---|---|---|---|---|---|---|---|---|
| Within 30 minutes | 3.93 (1.02) | 3.94 (1.01) | 3.85 (1.04) | 3.93 (1.00) | 3.69 (1.21) | 3.69 (1.22) | 3.70 (1.25) | 3.73 (1.22) |
| **Readiness to quit smoking** | | | | | | | | |
| Currently trying to quit | 3.86 (1.05) | 3.84 (1.06) | 3.78 (1.10) | 3.84 (1.08) | 3.54 (1.30) | 3.54 (1.17) | 3.54 (1.34) | 3.54 (1.30) |
| Will quit in the next month | 4.03 (0.96) | 4.09 (0.94) | 3.90 (0.96) | 4.01 (0.92) | 3.78 (1.12) | 3.84 (1.14) | 3.79 (1.17) | 3.87 (1.13) |
| **Motivation to quit smoking** | | | | | | | | |
| Low (1-3) | 3.58 (1.09) | 3.64 (1.07) | 3.54 (1.13) | 3.74 (1.06) | 3.19 (1.29) | 3.29 (1.27) | 3.14 (1.35) | 3.31 (1.29) |
| High (4-5) | 4.06 (0.97) | 4.03 (0.99) | 3.94 (1.01) | 3.95 (1.02) | 3.80 (1.19) | 3.78 (1.20) | 3.82 (1.22) | 3.79 (1.23) |
| **Psychological flexibility** (AAQ-II) | | | | | | | | |
| Flexible (7-27) | 3.88 (1.00) | 3.90 (1.00) | 3.80 (1.05) | 3.87 (1.03) | 3.58 (1.25) | 3.60 (1.24) | 3.58 (1.28) | 3.63 (1.26) |
| Inflexible (28-49) | 3.94 (1.09) | 3.91 (1.09) | 3.82 (1.11) | 3.91 (1.07) | 3.64 (1.27) | 3.65 (1.26) | 3.64 (1.35) | 3.64 (1.29) |

**Table 3**: Mixed models with main effects predicting message rating scores on dimensions content, design, coping, and quitting by message and participant / rater characteristics

| | Content | | | | Design | | | |
|---|---|---|---|---|---|---|---|---|
| | Unstandardized coefficient (b) | Standard error (SE) | T-value | p-value | Unstandardized coefficient (b) | Standard error (SE) | T-value | p-value |
| Message category: distraction (ref. acceptance) | 0.006 | (0.030) | 0.2 | 0.843 | 0.065 | (0.031) | 2.1 | 0.037 |
| Age | 0.020 | (0.013) | 1.5 | 0.128 | 0.026 | (0.013) | 1.9 | 0.054 |
| Male sex (ref. female) | 0.113 | (0.081) | 1.4 | 0.164 | 0.148 | (0.082) | 1.8 | 0.073 |
| SGM (ref. non-minority) | -0.189 | (0.106) | -1.8 | 0.074 | **-0.242*** | **(0.107)** | **-2.3** | **0.024** |
| Non-Hispanic Black (ref. NH White) | -0.093 | (0.097) | -1.0 | 0.341 | -0.123 | (0.099) | -1.3 | 0.210 |
| Hispanic (ref. NH White) | 0.110 | (0.101) | 1.1 | 0.274 | 0.063 | (0.102) | 0.6 | 0.540 |
| Other or multi-racial (ref. NH White) | 0.042 | (0.151) | 0.3 | 0.781 | -0.004 | (0.153) | 0.0 | 0.978 |
| Some college (ref. high school or less) | -0.173 | (0.094) | -1.8 | 0.065 | -0.179 | (0.095) | -1.9 | 0.061 |
| Bachelor's degree (ref. high school or less) | 0.095 | (0.104) | 0.9 | 0.365 | 0.111 | (0.106) | 1.1 | 0.293 |
| Graduate degree (ref. high school or less) | 0.070 | (0.131) | 0.5 | 0.595 | 0.182 | (0.133) | 1.4 | 0.171 |
| Daily smoking (ref. some days) | -0.153 | (0.087) | -1.8 | 0.078 | -0.148 | (0.088) | -1.7 | 0.093 |
| Time to first cigarette within 30 minutes (ref. after 30 mins) | -0.074 | (0.087) | -0.9 | 0.394 | -0.066 | (0.088) | -0.7 | 0.455 |
| Readiness to quit smoking within the next month (ref. currently trying to quit) | 0.082 | (0.091) | 0.9 | 0.367 | 0.036 | (0.092) | 0.4 | 0.694 |
| Motivation to quit smoking | **0.227*** | **(0.042)** | **5.5** | **0.000** | **0.151*** | **(0.042)** | **3.6** | **0.000** |
| Psychological flexibility (AAQ-II) | 0.000 | (0.005) | 0.0 | 0.971 | 0.000 | (0.005) | 0.1 | 0.925 |
| | | | | | | | | |
| | **Coping** | | | | **Quitting** | | | |
| Message category: distraction (ref. acceptance) | 0.024 | (0.034) | 0.7 | 0.485 | 0.038 | (0.034) | 1.1 | 0.268 |
| Age | 0.026 | (0.017) | 1.5 | 0.126 | 0.024 | (0.018) | 1.4 | 0.170 |
| Male sex (ref. female) | **0.330** | **(0.103)** | **3.2** | **0.001** | **0.308** | **(0.109)** | **2.8** | **0.005** |
| SGM (ref. non-minority) | -0.165 | (0.135) | -1.2 | 0.221 | -0.211 | (0.142) | -1.5 | 0.136 |
| Non-Hispanic Black (ref. NH White) | -0.102 | (0.124) | 0.8 | 0.408 | -0.147 | (0.130) | -1.1 | 0.259 |
| Hispanic (ref. NH White) | 0.184 | (0.128) | 1.4 | 0.151 | 0.188 | (0.135) | 1.4 | 0.164 |

| | | | | | | | | |
|---|---|---|---|---|---|---|---|---|
| Other or multi-racial (ref. NH White) | -0.143 | (0.192) | -0.7 | 0.458 | -0.223 | (0.203) | -1.1 | 0.272 |
| Some college (ref. high school or less) | -0.110 | (0.119) | -0.9 | 0.354 | -0.117 | (0.126) | -0.9 | 0.353 |
| Bachelor's degree (ref. high school or less) | 0.182 | (0.133) | 1.4 | 0.170 | 0.224 | (0.140) | 1.6 | 0.109 |
| Graduate degree (ref. high school or less) | **0.348*** | **(0.167)** | **2.1** | **0.037** | **0.456*** | **(0.176)** | **2.6** | **0.010** |
| Daily smoking (ref. some days) | **-0.295**** | **(0.110)** | **-2.7** | **0.007** | **-0.272*** | **(0.116)** | **-2.3** | **0.019** |
| Time to first cigarette within 30 minutes (ref. after 30 mins) | -0.079 | (0.110) | 0.7 | 0.476 | -0.044 | (0.116) | -0.4 | 0.704 |
| Readiness to quit smoking within the next month (ref. currently trying to quit) | 0.070 | (0.115) | 0.6 | 0.546 | 0.082 | (0.122) | 0.7 | 0.498 |
| Motivation to quit smoking | **0.223***** | **(0.053)** | **4.2** | **0.000** | **0.233***** | **(0.056)** | **4.2** | **0.000** |
| Psychological flexibility (AAQ-II) | 0.000 | (0.006) | 0.0 | 0.986 | -0.002 | (0.006) | -0.3 | 0.801 |

Note: NH = Non-Hispanic; AAQ-II = Acceptance and Action Questionnaire – version two;

*p<.05; **p<.01; ***p<.001

### Figures

| Distraction message | Acceptance message |
| --- | --- |
|   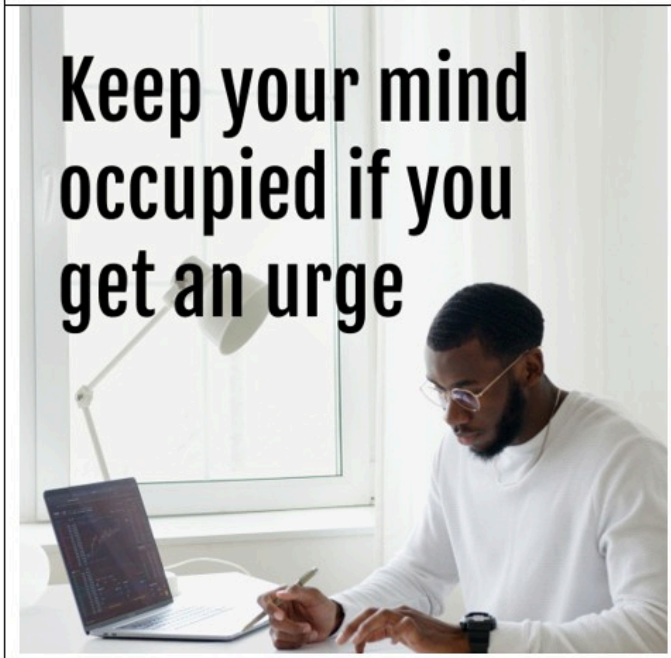 |   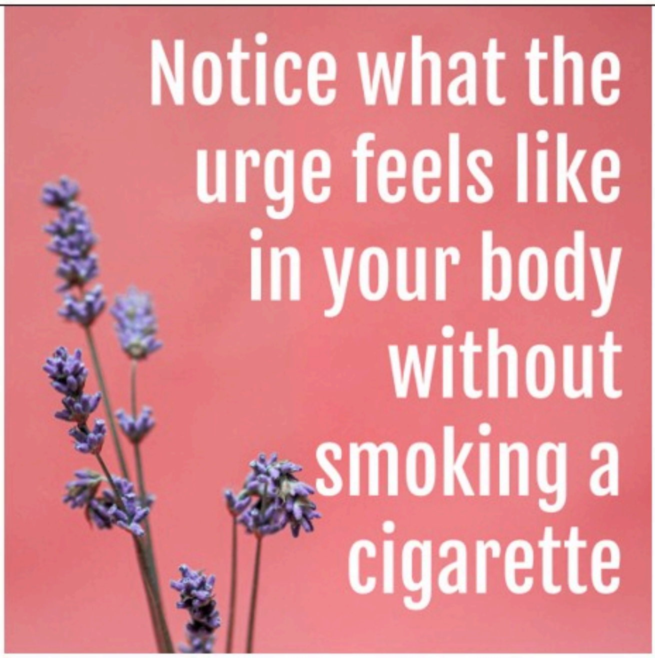 |
| Keep your mind occupied if you get an urge. Do a mental puzzle, make your next shopping list, read a book, write a poem…you might be surprised how well occupying your mind can get rid of urges. | Take a minute to notice what the urge feels like in your body without smoking a cigarette. Notice what is going on. See what happens. Does the urge pass? |

**Figure 1**

Intervention message examples for distraction and acceptance messages, including image and text content

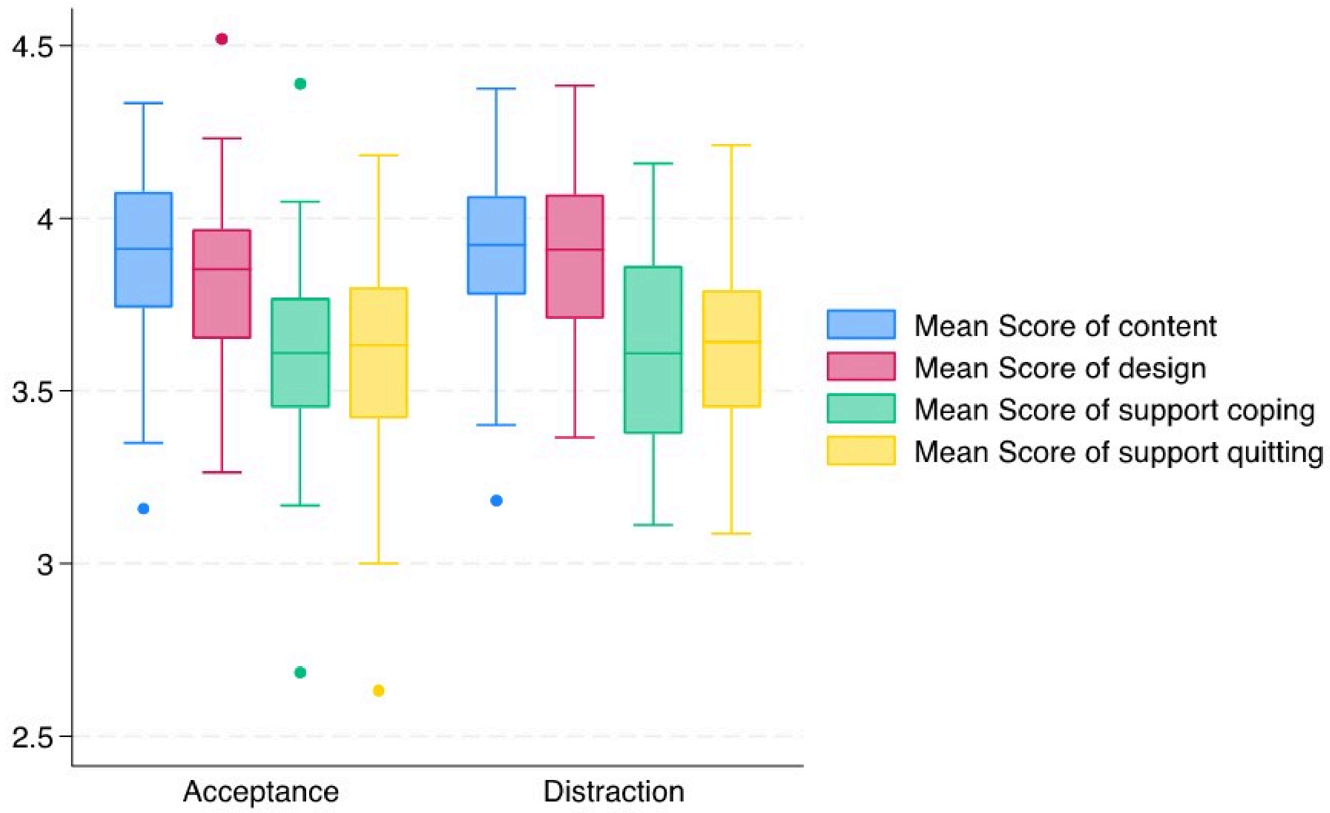

**Figure 2**

Acceptance and distraction messages and mean rating scores for each message on dimensions content, design, support for coping with smoking urges, and support for quitting smoking (median; interquartile range (IQR) Q1 to Q3; whiskers are lower and upper adjacent values, respectively)
